# Biological Therapy Leads to a Reduction in Systemic Inflammation but Leaves Serum Uric Acid Unmodified in Moderate-to-Severe Plaque Psoriasis: A Prospective Longitudinal Cohort Study

**DOI:** 10.3390/jcm15103817

**Published:** 2026-05-15

**Authors:** Larisa Ionela Suiu, Florentin Ananu Vreju, Adina Turcu-Stiolica, Loredana Elena Stoica, Mihai Turcu-Stiolica, Paulina Lucia Ciurea

**Affiliations:** 1 Doctoral School, University of Medicine and Pharmacy of Craiova, 200349 Craiova, Romania; larisasuiu10@yahoo.com; 2 Department of Rheumatology, University of Medicine and Pharmacy of Craiova, 200349 Craiova, Romania; paulina.ciurea@umfcv.ro; 3 Biostatistics Department, Faculty of Pharmacy, University of Medicine and Pharmacy of Craiova, 200349 Craiova, Romania; 4 Health Economics and Outcomes Research Department, Faculty of Medicine, “Iuliu Haţieganu” University of Medicine and Pharmacy Cluj-Napoca, Victor Babes Street No. 8, 400012 Cluj-Napoca, Romania; 5 Department of Dermatology, University of Medicine and Pharmacy of Craiova, 200349 Craiova, Romania; loredana.stoica@umfcv.ro; 6 Rheumatology Department, Emergency County Clinical Hospital of Craiova, 1 Tabaci Street, 200642 Craiova, Romania; mihaiturcu0812@gmail.com

**Keywords:** psoriasis, biological therapy, uric acid

## Abstract

**Background/Objective:** Psoriasis is a systemic inflammatory disease often associated with metabolic comorbidities, including hyperuricemia. While biological therapies effectively target inflammatory pathways, their specific impact on serum uric acid (SUA) levels remains debated. This study aimed to evaluate whether biological therapy, while reducing systemic inflammation, influences SUA levels in patients with moderate-to-severe plaque psoriasis. **Methods:** A prospective longitudinal cohort study was conducted involving 30 patients with moderate-to-severe plaque psoriasis. Patients received biological treatment (adalimumab, secukinumab or ustekinumab) and tsDMARDS (apremilast). Clinical severity was assessed using the Psoriasis Area and Severity Index (PASI). C-reactive protein (CRP), erythrocyte sedimentation rate (ESR), SUA levels and other laboratory markers were measured at baseline and after 48 weeks of therapy. **Results:** Biological therapy led to a significant reduction in PASI scores (from 21.6 ± 10.7 at baseline to 0.4 ± 0.86 after 48 weeks of therapy, *p* < 0.001), and CRP decreased from a median of 5.75 mg/L at baseline to 3.55 mg/L, *p* < 0.001. ESR also declined from 26.2 ± 11.4 mm/h to 19.0 ± 8.06 mm/h, *p* < 0.001. However, no statistically significant change was observed in mean SUA levels 5.49 ± 1.55 vs. 5.55 ± 1.60 mg/dL; *p* = 0.758. Subgroup analysis revealed that SUA levels remained stable regardless of the specific biological agent used or the degree of clinical improvement. **Conclusions:** Our findings suggest that while biological therapy is highly effective in controlling skin and systemic inflammation in psoriasis, it does not modify SUA levels. These results imply that hyperuricemia in psoriasis may be driven by metabolic factors independent of the primary inflammatory pathways targeted by current biologics.

## 1. Introduction

Psoriasis is a systemic, immune-mediated disease that is not limited to skin involvement. It is associated with a multitude of systemic inflammatory and metabolic factors. They can influence evolution, severity and response to treatment. Keratinocyte hyperproliferation and the presence of inflammation are influenced by the activity of interleukin 17 (IL-17) and interleukin 1 (IL-1) but also by the Th22-Th17 response [1]. The relationship of hyperuricemia with psoriasis is still controversial. Elevated serum uric acid (SUA) levels may be responsible for the exacerbation of skin lesions [2]. Hyperuricemia has a bidirectional correlation with psoriasis and psoriatic arthritis (PsA). Thus, patients with psoriasis and PsA frequently have elevated SUA levels. On the other hand, the presence of hyperuricemia itself is a risk factor for the development of psoriasis [3]. There is evidence that recognizes a genetic cause for the correlation of hyperuricemia with psoriasis in certain populations [4]. The importance of the presence of hyperuricemia in patients with psoriasis is even greater as it is a cardiovascular risk factor in these patients [5].

Long-term use of biological therapies, especially IL-17 inhibitors, in some patients with psoriasis may influence the occurrence of hyperuricemia [6]. On the other hand, in certain groups of patients, such as those aged >50 years and with a body mass index (BMI) < 24, therapy with IL-17A inhibitors reduces hyperuricemia [7].

Independent risk factors for hyperuricemia in patients with psoriasis are represented by young age, male gender, hypertension, obesity and hypertriglyceridemia [7]. Metabolic syndrome is frequently associated with psoriasis. Its components include abdominal obesity, dyslipidemia, hypertension and diabetes or insulin resistance. SUA, along with triglycerides (TG), cholesterol (Col), and low-density lipoprotein cholesterol (LDL), is a risk factor for the development of metabolic syndrome (Mets) in patients with psoriasis [8]. Obese or overweight patients with psoriasis have more frequent hyperuricemia than those with a normal body mass index [9].

The relationship between psoriasis and hyperuricemia is controversial. There are studies showing conflicting results regarding the risk factors for the development of hyperuricemia and the consequences of its presence in patients with psoriasis. Hyperuricemia may influence the response to treatment in patients with psoriasis. But on the other hand, certain therapies used to treat psoriasis may influence SUA levels. Overall, current evidence suggests that TNF-α inhibitors do not significantly reduce serum uric acid levels in patients with psoriasis. In contrast, data regarding IL-17 inhibitors remain inconsistent, with some studies reporting a potential urate-lowering effect, whereas others indicate an association with increased serum uric acid levels and possible hyperuricemia [6,7,10].

The primary aim of this study was to evaluate the effect of biological therapies on SUA levels in patients with moderate-to-severe plaque psoriasis over 48 weeks of treatment. A secondary aim was to assess the impact of these therapies on systemic inflammatory markers (CRP and ESR) and to explore the correlation between changes in disease severity, assessed using the Psoriasis Area and Severity Index (PASI), and changes in SUA, inflammatory markers, and other metabolic parameters.

## 2. Materials and Methods

### 2.1. Study Design

This study was conducted between October 2024 and October 2025. A total of 42 adult patients were included in the study, of which 30 met the participation criteria. Patients from the Dermatology Clinic, Craiova County Emergency Hospital, Romania, confirmed with psoriasis following skin biopsy were selected. Twelve patients were excluded: 2 pregnant patients, 4 patients with acute or chronic kidney disease responsible for increased uric acid, and 6 patients with biopsy results other than psoriasis.

The dynamics of skin lesion severity parameters and biological indices were monitored at baseline and at 48 weeks. Thus, we performed two calculations of disease activity, and two blood samples were taken.

The intensity of skin involvement was measured using the PASI, a standard method that analyzes both the affected body surface and the appearance of the lesions [11].

We monitored the values of metabolic markers represented by uric acid, considering hyperuricemia values > 7 mg/dL for men and >5 mg/dL for women, but also the values of serum lipids. We thus evaluated triglycerides and total cholesterol, as well as low-density lipoprotein (LDL) and high-density lipoprotein (HDL) fractions. Inflammatory markers were represented by erythrocyte sedimentation rate (ESR) and C-reactive protein (CRP). Glycemic status was also evaluated, as well as serum values of urea, creatinine, blood glucose, hemoglobin, leukocytes and platelets. We also recorded demographic data such as age and gender.

During the 48-week study, patients received therapy to treat the skin disease as follows: 11 patients received IL-17 inhibitors, 15 patients received IL-23 inhibitors, 2 patients received Tumor Necrosis Factor (TNF) inhibitors, and 2 received Phosphodiesterase 4 (PDE4) inhibitors. The first evaluation of the blood sample collection and skin lesion severity index was performed at baseline, before the start of therapy. The second evaluation of the same markers was performed after 48 weeks of psoriasis therapy.

### 2.2. Statistical Analysis

All statistical analyses were performed using software R (version 4.5.2, R Foundation for Statistical Computing, 2025, Vienna, Austria). Continuous variables were tested for normality using the Shapiro–Wilk test, given the sample size of *n* = 30. Normally distributed variables were expressed as mean ± standard deviation (SD), while non-normally distributed variables were expressed as median with interquartile range (IQR). Categorical variables were reported as absolute frequencies and percentages.

Within-group comparisons between baseline and 48-week follow-up measurements were performed using the paired-samples t-test for normally distributed variables and the Wilcoxon signed-rank test for non-normally distributed variables. Effect sizes were calculated using Cohen’s d, with values of 0.2, 0.5, and 0.8 interpreted as small, medium, and large effects, respectively.

To assess the relationship between the magnitude of change in disease severity and changes in inflammatory and metabolic parameters, Pearson or Spearman’s rank correlation coefficients were computed between delta values (defined as the difference between baseline and follow-up measurements) for DeltaPASI, DeltaCRP, DeltaESR, and DeltaUricAcid. Spearman’s method was selected given the non-normal distribution of several delta variables, as confirmed by the Shapiro–Wilk test and visual inspection of density plots. Correlation coefficients were interpreted according to the following thresholds: |r| < 0.2 negligible, 0.2–0.39 weak, 0.40–0.59 moderate, 0.60–0.79 strong, and ≥0.80 very strong.

For all analyses, a two-tailed *p*-value of less than 0.05 was considered statistically significant. Given the exploratory nature of the study and the limited sample size (*n* = 30), no correction for multiple comparisons was applied; all findings are therefore considered hypothesis-generating and should be interpreted with appropriate caution. No formal a priori sample size calculation was performed; this is acknowledged as a methodological limitation. A post hoc power analysis was performed using GPower 3.1.9.7 [12]. The findings of this study can be used to inform sample size calculations for future prospective, adequately powered investigations. All results are therefore interpreted with explicit acknowledgment of the risk of type II error.

## 3. Results

### 3.1. Characteristics of the Patients

This prospective, single-cohort, pre–post observational study included 30 patients with plaque psoriasis (50% male), with a mean age of 50.4 ± 14.2 years, followed over 48 weeks of biological therapy. Baseline clinical and laboratory characteristics are summarized in Table 1.

### 3.2. Clinical Response

**Figure 1:**
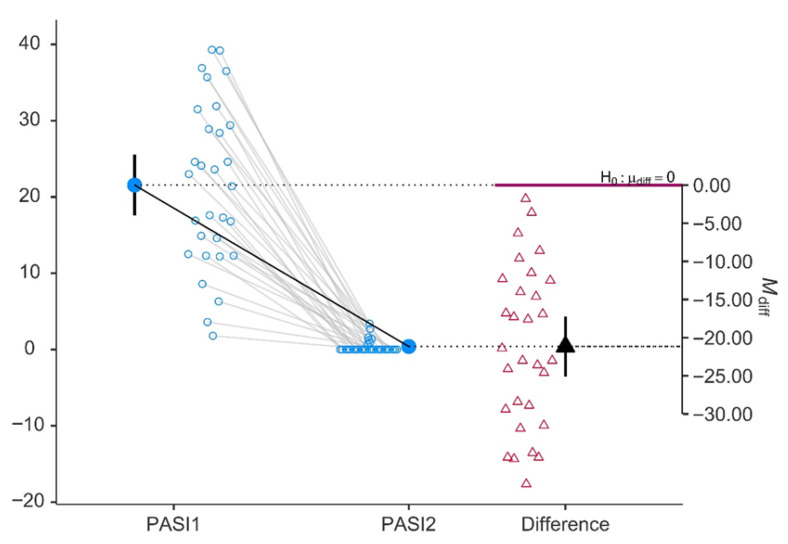
Psoriasis Area and Severity Index (PASI) baseline (PASI1) and after 48 weeks of therapy (PASI2). Light blue open circles on PASI1 and PASI2: individual patient scores at each time point. Each circle is one person’s measurement. Solid dark blue circles on PASI1 and PASI2: the group means at each time point. Gray thin lines connecting PASI1 to PASI2: each line links the same patient’s two measurements, showing the paired structure of the data. Black thick line between the two solid blue dots: connects the two group means, visually emphasizing the average change. Magenta open triangles in the “Difference” column: each patient’s individual change score (PASI2 − PASI1). Most are negative because most patients improved.

The most striking finding was the profound reduction in disease severity, as reflected by the PASI. Mean PASI decreased from 21.6 ± 10.7 at baseline to 0.4 ± 0.86 after 48 weeks of therapy (*p* < 0.001), corresponding to a large effect size (Cohen’s d = 0.937), as shown in Figure 1. This near-complete clinical response is visually corroborated by Figure 1, in which virtually all individual trajectories converge toward zero at follow-up, with the mean difference on the secondary axis approximating −21 points relative to the H_0_: μ_diff = 0 reference line.

### 3.3. Inflammatory Markers

**Figure 2:**
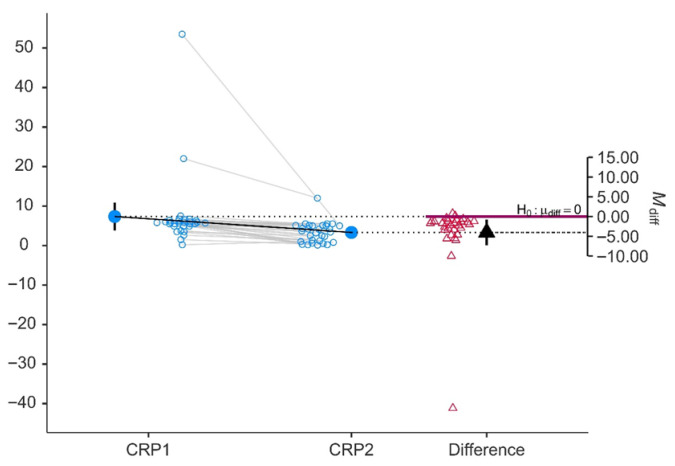
C-reactive protein (CRP) baseline (CRP1) and after 48 weeks of therapy (CRP2).

**Figure 3:**
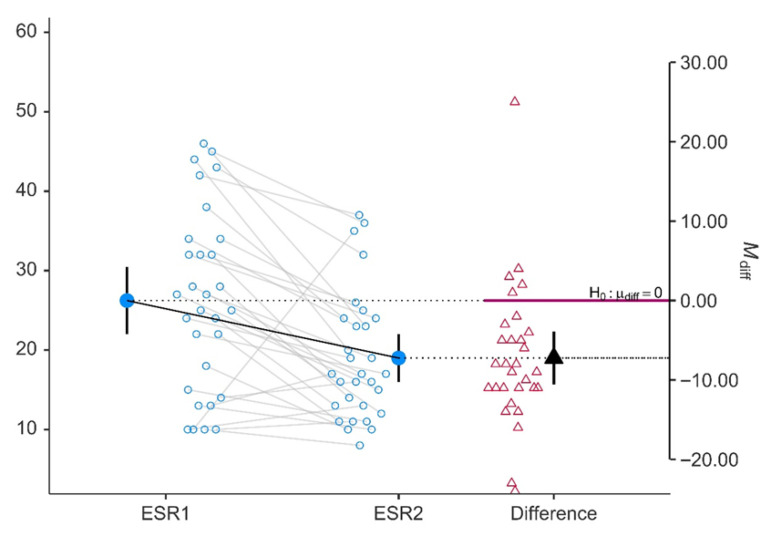
Erythrocyte sedimentation rate (ESR) baseline (ESR1) and after 48 weeks of therapy (ESR2).

Consistent with the clinical response, both acute-phase reactants demonstrated statistically significant and clinically meaningful reductions. CRP decreased from a median of 5.75 mg/L (IQR 4.9–6.27) at baseline to 3.55 mg/L (IQR 1.05–5.0) at follow-up (mean: 7.35 ± 9.38 vs. 3.32 ± 2.54; *p* < 0.001; Cohen’s d = 0.759), representing a large effect. ESR also declined significantly, from 26.2 ± 11.4 mm/h to 19.0 ± 8.06 mm/h (*p* < 0.001; Cohen’s d = 0.486), a medium effect size. Both paired plots (Figure 2 and Figure 3) demonstrate a consistent downward shift in the majority of individual patients, with the mean difference arrows positioned clearly below the null hypothesis line, confirming a systematic, non-random reduction in systemic inflammation.

### 3.4. Metabolic and Laboratory Parameters

SUA levels did not change significantly over the follow-up period (5.49 ± 1.55 vs. 5.55 ± 1.60 mg/dL; *p* = 0.758; Cohen’s d = 0.031), indicating that 48 weeks of biological therapy had no meaningful impact on uricemia at the group level. A post hoc power analysis for serum uric acid was performed using G*Power (version 3.1.9.7; Wilcoxon signed-rank test, matched pairs, two-tailed, α = 0.05, *n* = 30). The analysis was conducted using the “From differences” approach, with the mean of the within-subject difference scores (follow-up minus baseline) equal to 0.06 mg/dL and the standard deviation of the difference scores equal to 1.63 mg/dL, both derived directly from the raw dataset. The resulting standardized effect size was dz = 0.037. The achieved statistical power was 1 − β = 0.054 (5.4%), with a noncentrality parameter δ = 0.197, critical t = 2.050, and Df = 27.6. The type II error probability was β = 94.6%. This confirms that the study was critically underpowered for the serum uric acid outcome, and the non-significant result (*p* = 0.758) must be interpreted as entirely inconclusive rather than as evidence of the absence of an effect. Lipid parameters—total cholesterol (218 ± 43.7 vs. 219 ± 39.9 mg/dL; *p* = 0.823), LDL (121 ± 23.8 vs. 122 ± 28.2 mg/dL; *p* = 0.778), and triglycerides (137 ± 60.5 vs. 134 ± 55.4 mg/dL; *p* = 0.271)—remained stable throughout the observation period. HDL cholesterol showed a statistically significant but clinically negligible decrease (47.5 ± 9.12 vs. 46.9 ± 9.41 mg/dL; *p* = 0.024; Cohen’s d = 0.186), although the effect size was small (d = 0.186) and the absolute change of 0.6 mg/dL is below any clinically relevant threshold; this finding should not be interpreted as a clinically meaningful adverse drug effect. Fasting glucose declined modestly (103 ± 19.8 vs. 101 ± 25.3 mg/dL; *p* = 0.023; Cohen’s d = 0.27), reaching statistical significance; however, this effect is small and of uncertain clinical relevance, and may reflect regression toward the mean rather than a true drug effect. Renal function indices (urea and creatinine), complete blood count parameters (hemoglobin, leukocytes, and thrombocytes), and urea all remained within stable ranges without statistically significant variation.

**Figure 4:**
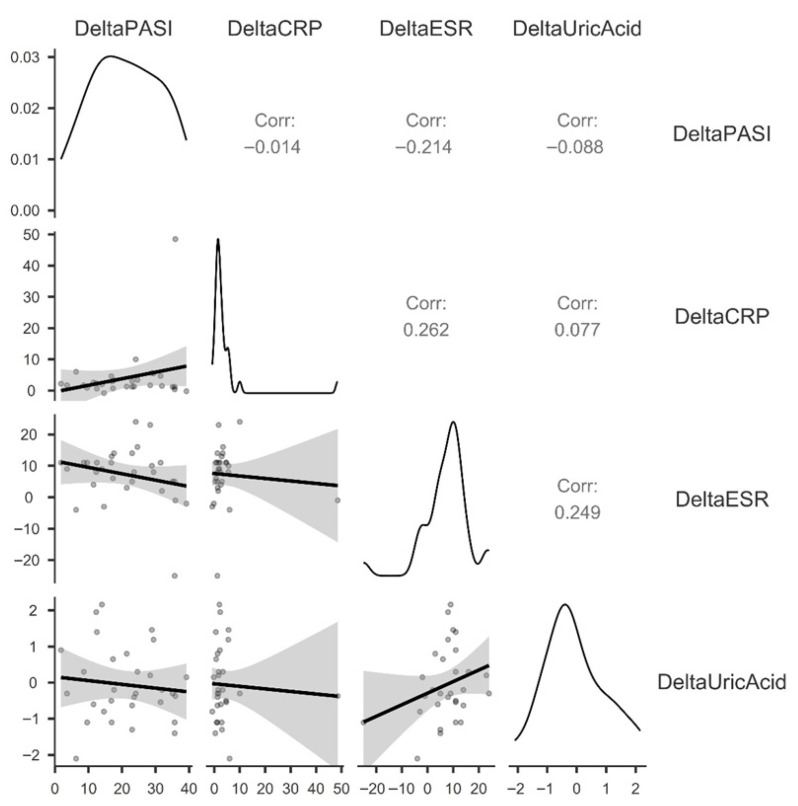
Correlation matrix between the magnitude of clinical response and uric acid change.

The correlation matrix of change scores (delta values = follow-up minus baseline) across the four key variables—DeltaPASI, DeltaCRP, DeltaESR, and DeltaUricAcid—is presented in Figure 4. Spearman or Pearson correlations (as appropriate given the distributional characteristics visible in the diagonal density plots) were computed for all six pairwise combinations.

The diagonal density plots reveal important features of the change score distributions. DeltaPASI displays a markedly right-skewed distribution consistent with a floor effect, as the majority of patients achieved near-complete skin clearance and therefore clustered near zero reduction, with a small number of larger responders pulling the tail rightward—reflecting the profound efficacy already documented in the paired analysis. DeltaCRP exhibits a highly leptokurtic distribution with an extreme right-sided outlier (approximately +50 units), indicative of one patient with an unusual CRP increase during follow-up that substantially influences this variable’s distributional properties. DeltaESR shows a broader, moderately dispersed distribution with a slight bimodal character, suggesting heterogeneity in inflammatory trajectory across patients. DeltaUricAcid is the most symmetric and approximately normally distributed of the four variables, centered near zero and ranging from approximately −2 to +2 mg/dL, which is visually consistent with the non-significant paired change observed in the primary analysis.

None of the six pairwise correlations reached the threshold of statistical significance (*p* > 0.05 for all, and the Pearson/Spearman correlation coefficients were less than 0.3 in module). The correlation between DeltaPASI and DeltaUricAcid was r = −0.088, indicating a negligible and non-significant inverse relationship between the magnitude of skin improvement and SUA change. DeltaPASI and DeltaCRP yielded r = −0.014, essentially zero, while DeltaPASI and DeltaESR produced the strongest association within this matrix at r = −0.214, a weak negative correlation that nonetheless failed to reach significance. Between the two inflammatory markers, DeltaCRP and DeltaESR showed a weak positive correlation (r = 0.262), suggesting modest convergent validity in tracking the inflammatory trajectory, though again non-significant. Crucially, both DeltaCRP and DeltaESR showed only negligible positive correlations with DeltaUricAcid (r = 0.077 and r = 0.249, respectively), indicating that the degree of inflammatory suppression during therapy did not meaningfully predict the direction or magnitude of SUA change.

## 4. Discussion

The principal finding of this study is that 48 weeks of sustained biological therapy in patients with moderate-to-severe plaque psoriasis was associated with significant reductions in both cutaneous disease activity and systemic inflammation, without a statistically significant change in SUA levels; however, with only 6.1% achieved power for this outcome, this null result is inconclusive.

In terms of the dissociation between skin clearance and uricemia, the near-complete resolution of PASI scores—with a large Cohen’s d of 0.937—confirms the well-established efficacy of biologic agents in controlling keratinocyte hyperproliferation and the IL-17/IL-23 inflammatory axis. Yet, despite this remarkable clinical response, SUA concentrations remained essentially unchanged (*p* = 0.758; Cohen’s d = 0.031). Xiangxian Liu et al. [6] also demonstrated that IL17 inhibitors had no effect on reducing SUA levels in patients with psoriasis. This dissociation is biologically plausible and has important implications. SUA is generated primarily through purine catabolism mediated by xanthine oxidase, a pathway that is only partially linked to the IL-17/Th17 inflammatory cascade central to plaque psoriasis pathophysiology. It is therefore conceivable that biological therapies, which act upstream of keratinocyte turnover, are insufficient to modulate the metabolic determinants of hyperuricemia, particularly in a population where independent risk factors—male sex, obesity, hypertension, dyslipidemia, and insulin resistance—are overrepresented. This is consistent with findings of Rui Huang et al. in a retrospective study from 2024 including data collected from multiple departments of a hospital in China on 1097 psoriasis patients, which showed that 87% of psoriasis patients had at least one comorbidity including hyperuricemia [13], although Minghui Hu et al. found no causal relationship between SUA and psoriasis [14]. Other groups suggest that hyperuricemia in psoriasis is not simply a consequence of epidermal cell turnover and nucleic acid release, but reflects a broader metabolic dysregulation that persists independently of skin clearance [15]. The authors of a 2021 meta-analysis [16] including 2082 patients concluded that there is a correlation between hyperuricemia and severe and moderate forms of psoriasis, unlike mild forms, where they found no correlation. This idea suggests that the presence of hyperuricemia may be influenced by cutaneous extension.

The significant reductions in CRP (Cohen’s d = 0.759) and ESR (Cohen’s d = 0.486) confirm that biological therapy effectively suppresses the systemic inflammatory burden of psoriasis, beyond its cutaneous manifestations. This is clinically relevant given that both CRP and ESR are independent cardiovascular risk markers, and patients with psoriasis carry a well-documented excess cardiovascular risk that is partially attributable to chronic low-grade systemic inflammation. The large effect size for CRP reduction is particularly noteworthy, as CRP is not only a surrogate of inflammation but also an active participant in endothelial dysfunction and atherogenesis. The consistent individual-level trajectories seen in Figure 2 and Figure 3—with the vast majority of patients showing directional improvement—underscore the robustness of this effect across the cohort. Rui Han et al. found in a retrospective study from 2025 that the measurement of serological parameters such as CRP and SUA, along with imaging methods, has predictive value for epicardial adipose tissue abnormalities in patients with psoriasis, being useful for the accurate diagnosis of cardiovascular comorbidities [17]. The association between CRP and heart rate in patients with psoriasis was proven by Paolo Compagnucci et al., who demonstrated in a multicenter study that CRP levels greater than 1 mg/dL increased the risk of recurrence of atrial tachyarrhythmia after ablation of fibrillation [18]. Serum CRP values have prognostic and diagnostic importance for cardiovascular comorbidities in patients with psoriasis. In addition, it is recognized that CRP has an impact on immune dysregulation, tissue damage, and disease progression [19].

The stability of total cholesterol, LDL, and triglycerides over 48 weeks is reassuring from a safety perspective, as certain systemic therapies carry adverse lipid profiles with chronic use. The statistically significant but trivial decline in HDL (Cohen’s d = 0.186; absolute difference 0.6 mg/dL) likely reflects biological noise rather than a clinically meaningful adverse effect, particularly given the absence of any corresponding LDL increase. The modest reduction in fasting glucose warrants cautious interpretation—while statistically significant, the Cohen’s d of 0.27 suggests a small effect, and this may represent regression toward the mean in a cohort with borderline dysglycemia at baseline, rather than a true metabolic benefit of therapy. Yong Chen et al. demonstrated that the use of biological therapies for the treatment of psoriasis does not influence lipid or glucose metabolism [20]. This evidence is consistent with our results. Tabra et al. [21] show the association of hyperuricemia with metabolic comorbidities, as demonstrated by our study. On the other hand, he claims that the skin damage severity index also correlates with increased SUA values, which contradicts the results of our study. Hagino et al. [22] demonstrated, contrary to our study, that certain biological therapies used in the treatment of psoriasis can reduce SUA and lipid levels. Furthermore, consistent with our results, the conclusions of a 2021 observational study revealed the correlation of SUA with metabolic dysfunctions but not the extension and severity of skin involvement [23].

Perhaps the most important clinical message of this study is the therapeutic gap it exposes. Patients with psoriasis who achieve full skin clearance may harbor persistent hyperuricemia that goes unmonitored and untreated, particularly as clinical attention shifts away from systemic parameters once cutaneous disease is controlled. Given that hyperuricemia is an independent cardiovascular risk factor in this population, and that it bidirectionally interacts with metabolic syndrome components—all of which were represented in this cohort—continued metabolic surveillance is essential even in biologic responders. Furthermore, since SUA, along with triglycerides, cholesterol, and LDL, is a recognized risk factor for the development of metabolic syndrome in psoriasis patients, the absence of uricemia reduction despite effective skin treatment reinforces the need for adjunctive metabolic management strategies, including dietary modification, xanthine oxidase inhibitors where appropriate, and lifestyle interventions targeting obesity and insulin resistance.

The near-zero correlation between DeltaPASI and DeltaUricAcid (r = −0.088) suggests that, in this exploratory cohort, the degree of cutaneous improvement—even when substantial (PASI reduction exceeding 20 points on average)—was not associated with how SUA levels evolved over the same period. While this observation is consistent with a model in which uricemia in psoriasis is not primarily driven by increased nucleic acid turnover from keratinocyte hyperproliferation, but rather by metabolic pathways such as purine dietary intake, renal urate excretion, xanthine oxidase activity, and insulin resistance-mediated urate retention, causal conclusions cannot be drawn from these correlational, underpowered data. These findings are hypothesis-generating and require confirmation in larger, adequately powered studies. The same conclusion was reached by Xin-Yu Gui et al. [24] in a descriptive cross-sectional study, in which SUA was not significantly associated with PASI values.

Regarding the weak inflammatory–uricemic axis, the correlations of DeltaCRP (r = 0.077) and DeltaESR (r = 0.249) with DeltaUricAcid were both positive and non-significant. The direction of both coefficients is, however, worthy of comment. A positive correlation between delta inflammatory markers and delta SUA would indicate that patients whose inflammation decreased the most also had the smallest reductions—or even increases—in SUA, while patients with less inflammatory suppression showed more SUA reduction. While neither coefficient is statistically robust at this sample size, the consistent positive directionality across both inflammatory markers hints at a possible dissociation between the anti-inflammatory and SUA-modifying effects of biological therapy or, alternatively, may reflect confounding by metabolic factors. The DeltaESR–DeltaUricAcid correlation of 0.249 is biologically worth noting: ESR is influenced not only by inflammation but also by serum proteins, lipid levels, and renal function—all of which interface with urate metabolism—potentially explaining its modestly stronger association with SUA change compared to CRP.

In terms of convergent validity of inflammatory markers, the positive correlation between DeltaCRP and DeltaESR (r = 0.262), while modest and non-significant, confirms that the two markers track in the same direction in most patients, providing some internal consistency. The fact that this correlation is not stronger likely reflects the well-known differences in the kinetics and determinants of the two markers: CRP is a highly specific acute-phase reactant with a short half-life driven predominantly by IL-6, whereas ESR is a more nonspecific integrative marker influenced by fibrinogen, immunoglobulins, and red cell morphology in addition to acute inflammation.

Regarding the weak inverse trend between DeltaPASI and DeltaESR, the correlation of r = −0.214 between DeltaPASI and DeltaESR, while non-significant, represents the strongest association within this matrix and is biologically plausible. It suggests a tendency—in the expected direction—for patients with greater PASI reductions to also show greater ESR decreases, consistent with the systemic anti-inflammatory consequences of skin disease control. The fact that this relationship is weak and non-significant likely reflects both the small sample size and the multifactorial nature of ESR, whose trajectory is influenced by factors beyond skin disease activity. Thus, Robert Olszewski et al. [25] have shown that the use of disease-modifying therapies that will reduce systemic inflammation also has a role in reducing the risk of cardiovascular events in psoriatic patients. They consider that hyperuricemia increases cardiovascular risk through secondary endothelial dysfunction even without the development of gout. A target SUA concentration should be established, and both non-pharmacological and pharmacological management should be recommended to reduce serum SUA concentration.

The correlation data between the magnitude of clinical response and SUA change reinforce the core clinical message: achieving excellent skin clearance with biological therapy does not confer protection against hyperuricemia or modify its trajectory. SUA management in patients with psoriasis must therefore be approached as an independent therapeutic target, governed by metabolic rather than dermatological considerations. Clinicians should not assume that patients who achieve PASI 90 or PASI 100 responses are metabolically protected. Longitudinal monitoring of serum uric acid, alongside lipid profile and glucose, remains mandatory throughout the course of biological therapy, irrespective of the degree of cutaneous response.

Psoriasis is a systemic disease that affects between 3 and 5% of the general population worldwide [24]. It is accompanied by numerous comorbidities [26]. Their presence has practical importance for the correct management of these patients. The association of hyperuricemia with psoriasis and psoriatic arthritis is documented, although there are recent studies that do not recognize this connection [27]. The role of hyperuricemia in psoriatic arthritis is complex, and the mechanisms underlying this link are incompletely explored. Although there are studies that do not recognize a causal relationship between serum SUA levels and psoriasis [27], others describe the frequent association of the two [28,29,30,31].

In addition to inflammation, traditional cardiovascular risk factors are markedly more prevalent in psoriatic patients. Obesity, hypertension, dyslipidemia, smoking, diabetes mellitus, and metabolic syndrome occur at higher frequencies in this population and may act synergistically with systemic inflammation to amplify cardiovascular risk. Importantly, conventional cardiovascular risk scores often underestimate the true vascular risk in psoriasis patients, particularly in severe disease and psoriatic arthritis [32,33,34,35].

There is evidence suggesting that psoriasis and cardiovascular disease share a common genetic background [36,37] and that targeted therapies for psoriasis may contribute to improved cardiovascular outcomes [33,37]. Inflammatory cytokines such as TNF-α, IL-17, and IL-23, which play key roles in the pathogenesis of psoriasis, are implicated in the development of cardiovascular disease through their contribution to vascular dysfunction and systemic inflammation. Consequently, biologic agents targeting these cytokines may provide benefits not only in controlling psoriasis but also in reducing cardiovascular risk [36,38].

An increasingly important aspect of cardiovascular risk assessment in psoriasis is the role of elevated serum uric acid [39]. There is evidence that hyperuricemia contributes to endothelial dysfunction and increased cardiovascular risk, but it remains debated whether it represents a direct causal factor or a marker of associated metabolic and inflammatory disorders [40,41].

In our study, the lack of correlation between PASI and SUA is consistent with the hypothesis that hyperuricemia in psoriasis is not merely a byproduct of epidermal cell turnover, but rather a reflection of systemic inflammatory burden and metabolic dysfunction. Furthermore, the correlation with metabolic markers suggests that metabolic syndrome comorbidities play a synergistic role in elevated serum urate levels, regardless of the cutaneous severity of the disease; however, this interpretation remains speculative in the absence of formal metabolic syndrome assessment in this cohort.

This study is limited by its relatively small sample size (*n* = 30) and the absence of a control group without biological therapy. The study also does not report baseline BMI or the prevalence of formal hyperuricemia (SUA > 7 mg/dL in men; >6 mg/dL in women), limiting the ability to stratify the SUA findings by metabolic phenotype. PASI is most useful in combination with other severity measures such as the Dermatology Life Quality Index (DLQI). PASI is more accurate in severe and moderate forms of psoriasis, while Body Surface Area (BSA) is indicated for mild forms; the use of multiple concurrent severity assessments would have improved measurement accuracy. Several additional limitations must be acknowledged. First, the study lacks a priori sample size justification; post hoc power analysis confirms that the study was substantially underpowered to detect small-to-moderate effects on uric acid, and the non-significant result cannot be interpreted as evidence of the absence of an effect. Second, all outcomes were assessed at only two time points (baseline and 48 weeks), without intermediate measurements; this design does not allow evaluation of early versus late treatment effects, the temporal dynamics of inflammatory versus metabolic responses, or transient changes in uric acid that may have occurred and resolved during follow-up—a fundamental limitation for a study described as longitudinal. Third, patients received four mechanistically distinct biological agents (IL-17 inhibitors, IL-23 inhibitors, TNF inhibitors, and PDE4 inhibitors), yet these were pooled into a single analysis; the differing pharmacological profiles and potential differential metabolic effects of these agents represent a significant source of heterogeneity that could not be adequately controlled, and agent-specific subgroup analyses were not powered to yield interpretable results. Fourth, key potential confounders were not collected or controlled, including body mass index, presence of metabolic syndrome components, concomitant medications (particularly uricosuric agents, diuretics, or drugs affecting purine metabolism), alcohol consumption, and smoking status, all of which are known to influence serum uric acid levels independently of psoriasis disease activity. Fifth, the correlation with PsA and obesity-related parameters could not be explored due to the absence of data on these variables; given the well-established independent associations of PsA and obesity with hyperuricemia, their inclusion in future studies is essential. Sixth, the single-arm pre–post design, without a comparator group receiving non-biological therapy or no treatment, precludes causal inference; observed changes in CRP and ESR cannot be attributed exclusively to biological therapy in the absence of a control arm. Diet or lifestyle data regarding smoking status or alcohol consumption are missing from our study. These could have influenced SUA values and our results. Future studies with larger cohorts, agent-specific analyses, and stratification by BMI, age, and comorbidity burden are needed to definitively characterize the relationship between biological therapy and uric acid metabolism in psoriasis.

## 5. Conclusions

This single-arm pre–post observational study of 30 patients with moderate-to-severe plaque psoriasis found that 48 weeks of biological therapy was associated with large, robustly detected reductions in PASI, ESR, and CRP, while SUA levels remained essentially unchanged; however, the post hoc power analysis revealed that the study had only 6% achieved power for the uric acid outcome, rendering this null result entirely inconclusive and insufficient to exclude a true biological effect. The observation that uricemia was unaffected despite profound cutaneous and inflammatory improvement is consistent with, but does not prove, the hypothesis that SUA in psoriasis is governed by metabolic pathways—including purine metabolism, renal urate handling, and insulin resistance—that may operate partially independently of the IL-17/IL-23 inflammatory axis targeted by current biological agents; this hypothesis requires dedicated investigation in adequately powered, prospective, agent-stratified studies with systematic collection of metabolic confounders. All findings in this study should be regarded as strictly hypothesis-generating, and the clinical recommendation that serum uric acid be monitored as an independent metabolic parameter in biologic responders—regardless of the degree of skin clearance achieved—while clinically plausible, awaits validation in larger controlled investigations before it can be incorporated into formal management guidelines.

## Figures and Tables

**Table 1:** Characteristics of the patients.

Characteristics	Baseline	After 48 Weeks of Therapy	*p*-Value	Cohen’s d
PASI	21.6 ± 10.7	0.4 ± 0.86	<0.001	0.937
	22.2 (13.0–29.3)	0		
Uric Acid, mg/dL	5.49 ± 1.55	5.55 ± 1.60	0.758	0.0305
	5.5 (4.23–6.97)	5.28 (4.21–7.16)		
Cholesterol, mg/dL	218 ± 43.7	219 ± 39.9	0.823	0.0262
	221 (187–256)	224 (193–245)		
LDL, mg/dL	121 ± 23.8	122 ± 28.2	0.778	0.0216
	120 (109–126)	117 (110–126)		
HDL, mg/dL	47.5 ± 9.12	46.9 ± 9.4	0.024	0.1864
	47.5 (45–52)	45 (43.3–51)		
TG, mg/dL	137 ± 60.5	134 ± 55.4	0.271	0.09
	135 (90.3–175)	123 (89.3–156)		
Hb, g/dL	13.8 ± 1.61	13.8 ± 1.82	0.87	0.02
	13.7 (13–15)	13.1 (12.2–15.5)		
L, µL	6711 ± 1392	6505 ± 1725	0.765	0.038
	6670 (5640–7740)	6550 (5688–7600)		
Tr, µL	256,100 ± 51,863 251,000 (234,500–283,750)	255,167 ± 73,382 253,000 (216,500–285,750)	0.865	0.028
Glucose, mg/dL	103 ± 19.8	101 ± 25.3	0.023	0.27
	99 (90–110)	92 (84.3–104)		
Urea, mg/dL	33.8 ± 7.51	32.3 ± 10.8	0.07	0.28
	34 (28.5–40.3)	29.5 (25.3–37.3)		
Creatinine, mg/dL	0.83 ± 0.19 0.82 (0.67–0.97)	0.79 ± 0.18 0.79 (0.66–0.89)	0.343	0.15
ESR, mm/h	26.2 ± 11.4	19 ± 8.06	<0.001	0.486
	26 (15.8–33.5)	17 (13–23.8)		
CRP, mg/L	7.35 ± 9.38	3.32 ± 2.54	<0.001	0.759
	5.75 (4.9–6.27)	3.55 (1.05–5)		

PASI—Psoriasis Area and Severity Index; LDL—low-density lipoprotein; HDL—high-density lipoprotein; TG—triglycerides, Hb—hemoglobin; L—leukocytes; Tr—platelets; ESR—erythrocyte sedimentation rate; CRP—C-reactive protein. All continuous variables are presented as mean ± standard deviation (SD) for normally distributed data, or as median (interquartile range, IQR) for non-normally distributed data, as determined by the Shapiro–Wilk test. The ± symbol denotes SD throughout.

## Data Availability

Research data are available at https://zenodo.org/records/18881712 (accessed on 1 April 2026).

## Abbreviations

The following abbreviations are used in this manuscript:

PASI	Psoriasis Area and Severity Index
CRP	C-Reactive Protein
ESR	Erythrocyte Sedimentation Rate
SUA	Uric Acid
IL	Interleukin
PsA	Psoriatic Arthritis
BMI	Body Mass Index
TG	Triglycerides
Col	Cholesterol
LDL	Low-Density Lipoprotein
Mets	Metabolic Syndrome
HDL	High-Density Lipoprotein
TNF	Tumor Necrosis Factor
PDE4	Phosphodiesterase 4
SD	Standard Deviation
IQR	Interquartile Range
Hb	Hemoglobin
L	Leukocyte
Tr	Platelets
DLQI	Dermatology Life Quality Index
BSA	Body Surface Area
